# Comparison of Intracellular Transcriptional Response of NHBE Cells to Infection with SARS-CoV-2 Washington and New York Strains

**DOI:** 10.3389/fcimb.2022.1009328

**Published:** 2022-09-20

**Authors:** Tiana M. Scott, Antonio Solis-Leal, J. Brandon Lopez, Richard A. Robison, Bradford K. Berges, Brett E. Pickett

**Affiliations:** ^1^ Department of Microbiology and Molecular Biology, Brigham Young University, Provo, UT, United States; ^2^ Population Health and Host-pathogen Interactions Programs, Texas Biomedical Research Institute, San Antonio, TX, United States

**Keywords:** SARS-CoV-2, COVID-19, data mining, bioinformatics, mechanism, virology, host response, host-virus

## Abstract

Severe acute respiratory syndrome coronavirus 2 (SARS-CoV-2) was first reported in Wuhan, China in December 2019 and caused a global pandemic resulting in millions of deaths and tens of millions of patients positive tests. While studies have shown a D614G mutation in the viral spike protein are more transmissible, the effects of this and other mutations on the host response, especially at the cellular level, are yet to be fully elucidated. In this experiment we infected normal human bronchial epithelial (NHBE) cells with the Washington (D614) strain or the New York (G614) strains of SARS-CoV-2. We generated RNA sequencing data at 6, 12, and 24 hours post-infection (hpi) to improve our understanding of how the intracellular host response differs between infections with these two strains. We analyzed these data with a bioinformatics pipeline that identifies differentially expressed genes (DEGs), enriched Gene Ontology (GO) terms and dysregulated signaling pathways. We detected over 2,000 DEGs, over 600 GO terms, and 29 affected pathways between the two infections. Many of these entities play a role in immune signaling and response. A comparison between strains and time points showed a higher similarity between matched time points than across different time points with the same strain in DEGs and affected pathways, but found more similarity between strains across different time points when looking at GO terms. A comparison of the affected pathways showed that the 24hpi samples of the New York strain were more similar to the 12hpi samples of the Washington strain, with a large number of pathways related to translation being inhibited in both strains. These results suggest that the various mutations contained in the genome of these two viral isolates may cause distinct effects on the host transcriptional response in infected host cells, especially relating to how quickly translation is dysregulated after infection. This comparison of the intracellular host response to infection with these two SARS-CoV-2 isolates suggest that some of the mechanisms associated with more severe disease from these viruses could include virus replication, metal ion usage, host translation shutoff, host transcript stability, and immune inhibition.

## Introduction

Near the end of 2019, a novel human coronavirus emerged in Wuhan, China (Zhou et al., 2020). This virus was subsequently named severe acute respiratory syndrome coronavirus 2 (SARS-CoV-2) and is the causative agent of coronavirus disease 2019 (COVID-19). Since its emergence, SARS-CoV-2 has caused a global pandemic that has cost millions of lives and disrupted many more (WHO, 2022). Even at early stages of the pandemic, it was noted that certain factors made individuals more susceptible to severe disease. Particularly, it was noted that the elderly and those with specific pre-existing conditions were more likely to develop severe symptoms than individuals of younger age groups (Sherwani and Khan, 2020; Lara et al., 2020). In addition, previous studies have observed that symptoms, severity of disease, and transmissibility can vary due to the viral strain or variant (Kim et al., 2020; Moghaddar et al., 2021; Radvak et al., 2021).

Mutations in the viral genome spontaneously occur during viral replication (Weber et al., 2021), which may result in viruses having an increase in overall fitness (Benedetti et al., 2020; Pachetti et al., 2020). If at least one of these mutations emerges in a viral genome, it could become a prominent strain in the host population until a new and more fit strain emerges (Baric, 2020; Plante et al., 2021). Coronaviruses have a proofreading mechanism, which results in a relatively low mutation rate for an RNA virus. Although the viral polymerase can fix many of the errors that occur during genome replication (Smith et al., 2013; Yan et al., 2021), mutations still occur, leading to the emergence of new SARS-CoV-2 strains, some of which have higher fitness than the original strain from 2019.

Recent studies have divided the different strains of SARS-CoV-2 into clades based on the presence of specific genetic mutations (Forster et al., 2020; Mercatelli and Giorgi, 2020). The D614G substitution in the viral spike protein has been the focus of many studies, and is characteristic of the G-clade (Volz et al.; Mercatelli and Giorgi, 2020; Zhang et al., 2020). This mutation has been linked to a higher transmissibility of the virus due to the stabilizing effect it has on the spike protein which is crucial for viral entry into the cell (Volz et al; Zhang et al., 2020). An analysis of the geographical prevalence of SARS-CoV-2 strains identified the G-clade in geographical regions that appeared to have higher levels of morbidity and mortality in the early stages of the pandemic. Specifically, areas such as the east coast of the United States, tended to have a higher percentage of G614 viruses than the west coast of the United States, which appeared to have lower mortality metrics (Brufsky, 2020). Since the first waves of infection, the G614 substitution spread rapidly and eventually became responsible for ~99% of the virus isolates collected from patients with severe or symptomatic disease (Mercatelli and Giorgi, 2020; Pandey et al., 2021; von Bartheld et al., 2021).

While there are many studies that have performed comparative genetic analyses on the different clades and strains of SARS-CoV-2 (Forster et al., 2020; Mercatelli and Giorgi, 2020), there are fewer studies that have looked at how these different strains affect the host intracellular transcriptional response. The aim of the current study was to use RNA sequencing and bioinformatics tools to analyze the transcriptome of normal human bronchial epithelial (NHBE) cells infected with two early isolates of SARS-CoV-2 that had differences in morbidity and mortality in human populations. The infected versus normal host transcriptomes of these cells were characterized at 6, 12, and 24 hours post-infection (hpi). Both strains were isolated from patient samples in early 2020. Our analysis found thousands of differentially expressed genes, hundreds of enriched gene ontology terms as well as dozens of affected signaling pathways. Overall, we found that the transcriptional changes in cells were similar at matched time points between cells infected with the different strains, but we also observed that the dysregulation of many pathways, particularly those involved in translation, were delayed in cells infected with the G614 strain. These differences in host response may at least partially account for some of the differences in the host response, and in the viral replication dynamics that were observed in geographical regions affected by early SARS-CoV-2 strains.

## Methods

Three biological replicates were performed in biosafety level three containment by growing three sets of NHBE cells to near confluency and separating them into two infected cultures and one mock-infected culture. Cell cultures were incubated for one hour with either the Washington (USA-WA1/2020) or New York (New York 1-PV08001/2020) isolate of SARS-CoV-2, which were obtained from BEI Resources, at a multiplicity of infection (MOI) of two. The media containing unattached virions was then removed, cells were washed three times with PBS and incubated in complete media at 37°CC with 5% CO_2_. Cells were harvested at 6, 12, and 24 hours post-infection (hpi). Mock-infected cells were incubated using the same protocol and reagents, and only lacked the presence of virus.

RNA was extracted from all samples using Trizol reagent according to the manufacturer’s protocol prior to quantification using a NanoDrop instrument. Reverse transcription of the RNA into cDNA was then performed prior to quantification, generation of Nextera XT sequencing libraries, and barcoding of samples for multiplexing. An Illumina NovaSeq instrument was then used to produce paired-end 150bp reads for each sample, with a mean of 27.2 million reads from each sample for downstream analysis.

The fastq files containing the RNA sequencing data were processed and analyzed using the existing Automated Reproducible MOdular Workflow for Preprocessing and Differential Analysis of RNA-seq Data (ARMOR) pipeline (Scott et al., 2021). Briefly, this software performs the following steps: generate read quality control metrics using fastQC (Website), trim sequencing adaptors and poor-quality regions of the reads with TrimGalore! (Babraham Bioinformatics, 2022), map and quantify reads to the human transcriptome using Salmon (Patro et al., 2017), calculate differentially expressed genes using edgeR (Robinson et al., 2010), predict differential transcript usage with DRIMseq (Nowicka and Robinson, 2016), and identify enriched Gene Ontology terms (The Gene Ontology Consortium, 2019) and Hallmark gene sets (Liberzon et al., 2015) using the Camera algorithm to adjust for inter-gene correlation (Durinck et al., 2005; Wu and Smyth, 2012).

The Ensembl identifiers for each of the differentially expressed genes, which were calculated with ARMOR, were then converted to the corresponding NCBI Entrez Gene identifiers through the BioMart database using R and Bioconductor (Huber et al., 2015) prior to calculating pathway enrichment with the Signaling Pathway Impact Analysis (SPIA) algorithm (Tarca et al., 2009). This pathway analysis algorithm applies a bootstrapping-based method to generate a null distribution that enables the calculation of robust statistics. The pathway information is derived from a combination of public databases including KEGG (Kanehisa and Goto, 2000), Reactome (Jassal et al., 2020), Panther (Mi et al., 2017), NCI (Schaefer et al., 2009), and BioCarta.

## Results

### Differentially expressed genes

We began by constructing *in silico* transcriptomic comparisons between infections for each virus isolate at each time point and the appropriate mock-infected controls (**Supplementary Tables S1**–**6**). This consisted of subjecting the quantified read mappings to a differential expression analysis using the edgeR algorithm. This approach provided us with a list of calculated differentially expressed genes for each time point for both virus strains. We then analyzed these lists to identify genes that were significantly affected (adjusted p-value <0.05). This process produced a list of 8,617 Ensembl gene identifiers that were significantly affected in at least one of the six examined conditions (two viruses at three time points), which correspond to 8,499 unique genes. Of these significant differentially expressed genes, we analyzed the ten genes that had the highest and lowest fold-change values (i.e. up- and down-regulated genes) for each condition and compared them to the significant genes identified in the other conditions. We constructed a table containing these top genes and how they are affected in the different strains (**Supplementary Table 7**).

Overall, we found the most similarity existing between matched time points of the two strains. We observed that cells infected by either virus at 6hpi shared 4 genes that were significantly up-regulated, (*IFITM10, TSEN, BDP1*, and *PPP1R11*). Of these genes only *BDP1* is significantly affected in another condition and it is down regulated at 24hpi for the Washington strain. Additionally at 6hpi in both strains, we observed seven genes were significantly down-regulated (*AC270230.1, RING1, PPP1R3B, PPP1R18, AC120057.2*, and *MCCC2*). *PPP1R18* was also down-regulated at 12hpi for the Washington strain. A list of the dysregulated genes at 6hpi for both strains, along with their fold change values and adjusted p-values, can be found in **Table 1**.

**Table 1:** Fold-change (case vs. control) and adjusted p-values for dysregulated genes at 6hpi for both SARS-CoV-2 isolates.

Gene		Washington 6hpi		New York 6hpi	
Ensembl ID	Gene Symbol	Log2 Fold-change	FDR adjusted p-value	Log2 Fold-change	FDR adjusted p-value
ENSG00000236560	*PPP1R11*	10.9	0.00236	10.5	0.00145
ENSG00000145734	*BDP1*	10.5	0.0321	9.95	0.0198
ENSG00000281618	*IFITM10*	8.49	0.0171	7.47	0.028
ENSG00000274129	*TSEN34*	5.09	0.0268	5.02	0.012
ENSG00000288461	*AC270230.1*	-10	0.0113	-10	0.00505
ENSG00000206287	*RING1*	-9.34	0.0157	-9.34	0.00717
ENSG00000285343	*PPP1R3B*	-9.34	0.0172	-9.34	0.00792
ENSG00000236428	*PPP1R18*	-9.27	0.0139	-9.27	0.00655
ENSG00000262526	*AC120057.2*	-8.2	0.0369	-8.2	0.0178
ENSG00000275300	*MCCC2*	-8.3	0.00485	-8.3	0.00222

The 12hpi conditions shared four genes (*DDX39B, AC139530.2, AC068533.4* and *GSDMC*) that were up-regulated and three genes (*NSF, GPANK1*, and *ZNF100*) that were down-regulated. Of the four up-regulated genes, one, *DDX39B*, was down-regulated at 6hpi for the Washington strain. Out of the three genes that were down-regulated, *NSF* was also down-regulated at 24hpi for the Washington strain, and *ZNF100* was up-regulated at 6hpi for the New York strain. This set of genes includes several that are thought to play roles in the immune response such as *GPANK1, DDX39B*, and *GSDMC.* A list of the dysregulated genes at 12hpi for both strains, along with their fold change values and adjusted p-values, can be found in **Table 2**.

**Table 2:** Fold-change (case vs. control) and adjusted p-values for dysregulated genes at 12hpi for both SARS-CoV-2 isolates.

Gene		Washington 12hpi		New York 12hpi	
Ensembl ID	Gene Symbol	Log2 Fold-change	FDR adjusted p-value	Log2 Fold-change	FDR adjusted p-value
ENSG00000230624	*DDX39B*	8.43	0.0256	7.24	0.0421
ENSG00000262660	*AC139530.2*	8.47	0.0124	6.35	0.0377
ENSG00000249319	*AC068533.4*	6.22	0.0131	5.23	0.0243
ENSG00000147697	*GSDMC*	2.86	0.00148	2.24	0.00959
ENSG00000274746	*ZNF100*	-8.91	0.011	-8.91	0.0076
ENSG00000278174	*NSF*	-9.49	0.00881	-7.99	0.0117
ENSG00000236011	*GPANK1*	-8.44	0.00778	-8.44	0.0062

The 24hpi conditions shared eight up-regulated genes (*FAM189B, AGPAT1, PPP1R3B, PRR3, ZBED6, SLC43A2, MAML1* and *AC020907.6*) and nine down-regulated genes (*SLC30A8, AC022149.1, RSPH14, AGXT, MAP3K15, AL136295.4, AL691477.1, AL358113.1* and *ARHGAP23*). One of the up-regulated genes, *AGPAT1*, was significantly down-regulated during infection with the New York strain at 12hpi. Another of the up-regulated genes, *PPP1R3B* was up-regulated at both 6hpi and 12hpi during infection with the New York strain. While the gene *PPP1R3B* is also seen to be down-regulated at 6hpi, it is categorized under a different Ensembl ID number and is therefore analyzed separately. None of the down-regulated genes were significantly affected at any other time point. A list of the dysregulated genes at 24hpi for both strains, along with their fold change values and adjusted p-values, can be found in **Table 3**.

**Table 3:** Fold-change (case vs. control) and adjusted p-values for dysregulated genes at 24hpi for both SARS-CoV-2 isolates.

Gene		Washington 24hpi		New York 24hpi	
Ensembl ID	Gene Symbol	Log2 Fold-change	FDR adjusted p-value	Log2 Fold-change	FDR adjusted p-value
ENSG00000160767	*FAM189B*	11.2	0.00162	11.9	0.000179
ENSG00000206324	*AGPAT1*	11.8	4.45E-07	11.9	2.46E-07
ENSG00000229202	*PRR3*	11.6	1.62E-05	11.4	4.66E-05
ENSG00000257315	*ZBED6*	10.9	0.0219	11.7	0.00321
ENSG00000173281	*PPP1R3B*	12.8	2.87E-05	12.5	0.000109
ENSG00000278550	*SLC43A2*	11	0.00538	11	0.00697
ENSG00000283780	*MAML1*	11.5	0.0353	11.6	0.0357
ENSG00000285526	*AC020907.6*	10.8	0.0365	10.9	0.0353
ENSG00000100218	*RSPH14*	-11.1	2.16E-05	-6.3	0.000209
ENSG00000164756	*SLC30A8*	-11.7	9.07E-06	-7.07	4.82E-05
ENSG00000172482	*AGXT*	-2.28	0.0456	-9.37	0.000182
ENSG00000180815	*MAP3K15*	-10	0.000305	-10	0.000222
ENSG00000259522	*AL136295.4*	-10.5	8.32E-05	-10.5	4.28E-05
ENSG00000273291	*AC092042.3*	-10.8	0.00164	-10.8	0.00158
ENSG00000273780	*ARHGAP23*	-11	0.0364	-11	0.0408
ENSG00000280249	*AL691477.1*	-4.06	0.000224	-10.5	4.51E-06
ENSG00000285130	*AL358113.1*	-11.5	0.0012	-3.53	0.0357

### GO Terms

In order to make the analysis of the differentially expressed genes easier to interpret, we used the camera algorithm to analyze the significantly enriched Gene Ontology (GO) terms and Hallmark gene sets for infection with each isolate and time point (**Supplementary Tables S8**–**13**; **Supplementary Figure S1**). These terms and gene sets enable us to better understand some of the mechanistic biological processes and molecular functions that were over-represented in the lists of differentially expressed genes. We identified 211 terms that were significantly enriched in at least one condition. While there were several terms that were significantly enriched at 6hpi and 12hpi with either strain, there were no significantly enriched terms at 24hpi for either virus. Of these 211 terms we analyzed the ten most significant positively- and negatively-regulated terms for each condition, or all available terms if there were less than ten, and compared these terms to those that were identified as significant in the other conditions. A list of these terms and their adjusted p-values can be found in **Table 4**.

**Table 4:** Directionality and adjusted p-values for enriched terms across multiple time points and SARS-CoV-2 isolates.

Database	Term	New York 6hpi		Washington 6hpi		New York 12hpi		Washington 12hpi	
		FDR adjusted p-value	Direction	FDR adjusted p-value	Direction	FDR adjusted p-value	Direction	FDR adjusted p-value	Direction
GO	Detoxification of inorganic compound	0.00043	Down	0.000104	Down	0.000353	Down	2.05E-06	Down
Reactome	Metallothioneins bind metal	0.00126	Down	0.000104	Down	0.001844	Down	1.70E-05	Down
Reactome	Response to metal ions	0.0086	Down	0.000813	Down	0.00269	Down	6.25E-05	Down
Hallmark	Amit serum response 120 MCF10A	0.001	Up	0.018998	Up	0.01118	Up		
Hallmark	Dazard UV response cluster G28	0.00043	Up	0.000602	Up	0.042113	Up		
GO	Lung vasculature development	0.00043	Up	0.000813	Up	0.033232	Up		
Hallmark	Lin silenced by tumor microenvironment	0.00043	Up	0.003791	Up	0.0189	Up		
GO	Calcium independent cell-cell adhesion *via* plasma membrane cell adhesion molecules	0.00341	Up			0.00403	Up	0.014062	Up
Reactome	Tight junction interactions	0.01483	Up			0.01118	Up	0.034701	Up
GO	Cellular response to zinc ion			0.009728	Down	0.033232	Down	0.009274	Down
Hallmark	Zheng response to arsenite up			0.049621	Down	0.013182	Down	0.000588	Down
Hallmark	Blanco Melo Beta interferon treated bronchial epithelial cells up	0.00341	Up			0.013182	Up		
Hallmark	Blanco Melo COVID-19 bronchial epithelial cells SARS-Cov-2 infection up	0.00043	Up			0.033232	Up		
Hallmark	Blanco Melo COVID-19 SARS-CoV-2 infection Calu-3 cells up	0.00643	Up			0.006878	Up		
Hallmark	Blanco Melo human parainfluenza virus 3 infection A549 cells up	0.00045	Up			0.027899	Up		
GO	CXCR chemokine receptor binding	0.01568	Up			0.013182	Up		
Hallmark	Hecker IFNB1 targets	0.00043	Up			0.009306	Up		
Hallmark	Moserle IFNA response	0.00341	Up			0.003021	Up		
Reactome	Cholesterol biosynthesis	0.00318	Down			0.003408	Down		
GO	Farnesyl diphosphate metabolic process	0.01853	Down			0.01118	Down		
Hallmark	Schmidt POR targets in limb bud up	0.00064	Down			0.000353	Down		
Hallmark	Weng POR targets global up	0.03123	Down			0.00687	Down		
WP	Cholesterol biosynthesis pathway	0.00047	Down			0.000353	Down		
WP	Mevalonate pathway	0.00142	Down			0.001967	Down		
Hallmark	Horton SREBF targets	0.00085	Down			0.000618	Down		
Reactome	Interleukin 10 signaling	0.00017	Up	0.000529	Up				
GO	Positive regulation of immature T-cell proliferation	0.00081	Up	0.002769	Up				
GO	Regulation of adiponectin secretion	0.00498	Up	0.010201	Up				
Hallmark	Onder CDH1 targets 3 down	0.00028	Up	0.010201	Up				
Hallmark	Dazard UV response cluster G2	0.00251	Up	0.003791	Up				
Hallmark	Liang silenced by methylation down	0.01779	Down					0.02866	Up
Hallmark	Browne interferon responsive genes	0.00043	Up						
Hallmark	Croonquist IL6 deprivation down	0.00111	Down						
Hallmark	Blanco Melo bronchial epithelial cells Influenza A delNS1 infection down	0.00043	Down						
Hallmark	Graham normal quiescent vs normal dividing down	0.0012	Down						
Hallmark	Kang doxorubicin resistance up	0.00142	Down						
Hallmark	Rosty cervical cancer prolieration cluster	0.0012	Down						
GO	Gas transport			0.009248	Up				
Hallmark	Shin B-cell lymphoma cluster 6			0.002769	Up				
Hallmark	Amit delayed early genes					0.014027	Up		
Hallmark	Bowie response to tamoxifen					0.011965	Up		
GO	Regulation of cellular pH reduction							0.037071	Up
HP	Orthokeratosis							0.009274	Up
Hallmark	Montero thyroid cancer poor survival up							0.009274	Up
Hallmark	Fung IL2 signaling 2							0.00375	Down
GO	Box C/D snoRNP complex							0.002785	Down
GO	Small subunit processome							0.013573	Down
Hallmark	Nadella PRKAR1A targets down							0.009274	Down
Reactome	rRNA modification in the nucleus and cytosol							0.021817	Down

This analysis produced a shorter list of 49 terms, with three being negatively regulated in all four conditions (both viruses, at 6hpi and 12hpi). These terms included the “detoxification of inorganic compound”, “metallothioneins bind metals”, and “response to metal ions”. Two additional terms, “cellular response to zinc ion” and “response to arsenite”, were negatively regulated in each condition except for 6hpi with the New York strain. Several terms were positively regulated in all conditions except at 12hpi in the Washington strain, which include “UV response cluster G28”, “serum response 120 MCF10A”, “lung vasculature development”, and “silenced by tumor microenvironment”. Additional terms that were positively regulated in all conditions besides 6hpi in the Washington strain include “tight junction interactions” and “calcium independent cell-cell adhesion *via* plasma membrane cell adhesion molecules”. Many of these terms are involved in how the body deals with and responds to metal ions. These terms, and their respective adjusted p-values, can be found in **Table 5**. On further comparison of the dysregulated terms we also found that there were more similarities between different time points for the same strain than for matched time points of the two different strains.

**Table 5:** Directionality and adjusted p-values for enriched terms associated with metal ions across multiple time points and SARS-CoV-2 isolates.

Database	Term	New York 6hpi		Washington 6hpi		New York 12hpi		Washington 12hpi	
		FDR adjusted p-value	Direction	FDR adjusted p-value	Direction	FDR adjusted p-value	Direction	FDR adjusted p-value	Direction
GO	Detoxification of inorgainc compound	0.00043	Down	0.000104	Down	0.000353	Down	2.05E-06	Down
Reactome	Metallothioneins bind metal	0.00126	Down	0.000104	Down	0.001844	Down	1.70E-05	Down
Reactome	Response to metal ions	0.0086	Down	0.000813	Down	0.00269	Down	6.25E-05	Down
Hallmark	Amit serum response 120 MCF10A	0.001	Up	0.018998	Up	0.01118	Up		
Hallmark	Dazard UV response cluster G28	0.00043	Up	0.000602	Up	0.042113	Up		
GO	Lung vasculature development	0.00043	Up	0.000813	Up	0.033232	Up		
Hallmark	Lin silenced by tumor microenvironment	0.00043	Up	0.003791	Up	0.0189	Up		
GO	Calcium independent cell-cell adhesion *via* plasma membrane cell adhesion molecules	0.00341	Up			0.00403	Up	0.014062	Up
Reactome	Tight junction interactions	0.01483	Up			0.01118	Up	0.034701	Up
GO	Cellular response to zinc ion			0.009728	Down	0.033232	Down	0.009274	Down
Hallmark	Zheng response to arsentite up			0.049621	Down	0.013182	Down	0.000588	Down

At 6hpi and 12hpi with the New York strain there are seven shared terms that are negatively regulated. These terms include “farnesyl diphosphate metabolic process,” “cholesterol biosynthesis”, “mevalonate pathway”, “SREBF targets”, “POR targets in limb bud”, and “POR targets global.” Many of these negatively regulated terms play a role in metabolism, specifically involving cholesterol and its precursors. There are also seven terms that are positively regulated at 6hpi and 12hpi with the New York strain. This includes “CXCR chemokine receptor binding,” “IFNA response,” “IFNB1 targets,” “Upregulated in beta interferon treated bronchial epithelial cells,” “Upregulated in COVID19 bronchial epithelial cells,” “Upregulated in COVID19 SARS-CoV-2 infection CALU3 cells,” and “Upregulated in human parainfluenza virus 3 infection A594 cells.” These positively regulated terms are mainly related to the immune system or represent groups of genes noted to be affected after viral infections. The adjusted p-values for these terms can be found in **Table 6**.

**Table 6:** Directionality and adjusted p-values for enriched terms associated with host immune response across multiple time points with the New York isolate of SARS-CoV-2.

Database	Term	New York 6hpi		New York 12hpi	
		FDR adjusted p-value	Direction	FDR adjusted p-value	Direction
Hallmark	Blanco Melo Beta interferon treated bronchial epithelial cells up	0.00341	Up	0.013182	Up
Hallmark	Blanco Melo COVID-19 bronchial epithelial cells SARS-Cov-2 infection up	0.00043	Up	0.033232	Up
Hallmark	Blanco Melo COVID-19 SARS-CoV-2 infection Calu-3 cells up	0.00643	Up	0.006878	Up
Hallmark	Blanco Melo human parainfluenza virus 3 infection A549 cells up	0.00045	Up	0.027899	Up
GO	CXCR chemokine receptor binding	0.01568	Up	0.013182	Up
Hallmark	Hecker IFNB1 targets	0.00043	Up	0.009306	Up
Hallmark	Moserle IFNA response	0.00341	Up	0.003021	Up
Reactome	Cholesterol biosynthesis	0.00318	Down	0.003408	Down
GO	Farnesyl diphosphate metabolic process	0.01853	Down	0.01118	Down
Hallmark	Schmidt POR targets in limb bud up	0.00064	Down	0.000353	Down
Hallmark	Weng POR targets global up	0.03123	Down	0.00687	Down
WP	Cholesterol biosynthesis pathway	0.00047	Down	0.000353	Down
WP	Mevalonate pathway	0.00142	Down	0.001967	Down
Hallmark	Horton SREBF targets	0.00085	Down	0.000618	Down

Interestingly, there are no terms that are exclusively shared at 6hpi and 12hpi with the Washington strain and there are no terms exclusively shared at 12hpi with either virus. There are, however, five terms that are positively regulated at 6hpi with both the New York and the Washington strain. These terms include “UV response cluster G2,” “Positive regulation of immature T-cell proliferation,” “regulation of adiponectin secretion,” “CDH1 targets,” and “Interleukin 10 signaling.” Similar to the terms that were affected at 6hpi and 12hpi with the New York strain, these terms are mostly related to the immune system. A list of these terms and their respective adjusted p-values can be found in **Table 7**.

**Table 7:** Directionality and adjusted p-values for enriched terms identified at 6hpi with either the New York or Washington isolate of SARS-CoV-2.

Database	Term	New York 6hpi		Washington 6hpi	
		FDR adjusted p-value	Direction	FDR adjusted p-value	Direction
Reactome	Interleukin 10 signaling	0.00017	Up	0.000529	Up
GO	Positive regulation of immature T-cell proliferation	0.00081	Up	0.002769	Up
GO	Regulation of adiponectin secretion	0.00498	Up	0.010201	Up
Hallmark	Onder CDH1 targets 3 down	0.00028	Up	0.010201	Up
Hallmark	Dazard UV response cluster G2	0.00251	Up	0.003791	Up

### Intracellular signaling pathways

To determine what intracellular pathways were significantly affected by the viral infection, we used the differentially expressed genes that we previously identified as input for the SPIA algorithm. This method generates a null distribution for each of over 1500 pathways that are present in five public pathway databases, which facilitates subsequent statistical calculations. These signaling pathways include manually curated protein-protein interactions, particularly those that convey signals from receptors on the cell surface to the nucleus. We performed this analysis for infections with each viral isolate and time point (**Supplementary Tables S14**–**19**).

This analysis yielded 29 significantly affected pathways that were present in at least one of the conditions. A list of these pathways and whether they were activated or inhibited in each condition is shown in **Table 8**. This included many pathways that are involved in the process of translation as well as rRNA processing. We manually reviewed these pathways to determine those that were shared between time points and conditions. We found that the “plasminogen activating cascade” signaling pathway was shared between the 6hpi time points of infection with the New York and Washington strains. This pathway was inhibited in both conditions and is involved in cell invasion, fibrin degradation, and matrix turnover (Patrizia Stoppelli, 2013). The 12hpi time points shared no significantly affected pathways and the 24hpi time points shared two significantly affected pathways. Infections with either virus inhibited the olfactory transduction pathway and activated the platelet derived growth factor (PDGFR)-beta signaling pathway.

**Table 8:** All significant positively (+) or negatively (-) dysregulated intracellular signaling pathways across multiple time points with either SARS-CoV-2 isolate.

Pathway name	NY 6hr	NY 12hr	NY 24hr	WA 6hr	WA 12hr	WA 24hr
Cell cycle	0.022257 (-)					
Keratinization	0.001558 (+)	0.017467 (+)				
Stabilization and expansion of the E-cadherin adherens junction	0.017463 (+)					
Alzheimer disease-presenilin pathway	0.004439 (+)					
Plasminogen activating cascade	0.007253 (-)	0.034549 (-)		0.036979 (-)		
Integrin signalling pathway	0.007253 (+)					
Regulation of actin cytoskeleton		0.035812 (-)	0.010134 (+)			
Signaling by TGF-beta Receptor Complex		0.00051 (+)				
mTOR signaling pathway		0.049417 (-)				
Olfactory transduction			0.000384 (-)			1.58E-05 (-)
Peptide chain elongation			4.76E-09 (-)		1.62E-08 (-)	
Nonsense-Mediated Decay (NMD)			4.76E-09 (-)		1.39E-05 (-)	
Nonsense Mediated Decay (NMD) enhanced by the Exon Junction Complex (EJC)			4.76E-09 (-)		1.39E-05 (-)	
Eukaryotic Translation Elongation			4.76E-09 (-)		3.23E-09 (-)	
Eukaryotic Translation Termination			1.63E-06 (-)		1.63E-06 (-)	
Translation			7.03E-05 (-)		0.000295 (-)	
SRP-dependent cotranslational protein targeting to membrane			0.000171 (+)		0.000204 (-)	
GTP hydrolysis and joining of the 60S ribosomal subunit			0.000417 (-)		0.000238 (-)	
Eukaryotic Translation Initiation			0.001691 (-)		0.000295 (-)	
Cap-dependent Translation Initiation			0.001691 (-)		0.000295 (-)	
rRNA processing in the nucleus and cytosol			0.004313 (-)		3.78E-13 (-)	
PDGFR-beta signaling pathway			0.010419 (+)			0.003059 (+)
Apoptosis				1.59E-05 (+)		
Amyotrophic lateral sclerosis (ALS)				0.001498 (+)		
MAPK signaling pathway				0.002158 (+)		
Hippo signaling pathway					0.006153 (+)	
Major pathway of rRNA processing in the nucleolus and cytosol					3.32E-13 (-)	
rRNA processing					4.45E-13 (-)	
Gene Expression					0.000162 (-)	

NY, New York; WA, Washington; “+”, Activated; “-”, Inhibited.

We also compared the pathways that were dysregulated at different time points during infection with the same virus. We found that the 6hpi and 12hpi time points for infection with the New York strain shared two dysregulated pathways including the activated “keratinization”pathway, and the inhibited “plasminogen activating cascade”. The 12hpi and 24hpi time points both showed dysregulation in the “actin cytoskeleton” pathway during infection with the NY strain; however, this pathway was affected in different directions–the 12hpi condition being inhibited and the 24hpi condition being activated. We observed no significantly dysregulated signaling pathways for the Washington strain at multiple time points.

The conditions that shared the most dysregulated pathways were the 24hpi New York condition and the 12hpi Washington condition. They shared a total of 10 dysregulated pathways which mainly dealt with translation. These 10 pathways were all inhibited in both conditions. Infection with either viral strain led to dysregulation in the “SRP-dependent cotranslational protein targeting to membrane” pathway but in different directions with the New York condition being activated and the Washington condition being inhibited.

## Discussion

The goal of the current study was to generate and analyze RNA-sequencing data to compare the underlying mechanism(s) associated with infection at three time points (6, 12, and 24 hpi) with two SARS-CoV-2 isolates that were collected during the early stages of the pandemic. We found thousands of differentially expressed genes, hundreds of enriched functional terms, and various signaling pathways that provide additional insight into why one of these strains may result in higher pathogenesis and mortality in the human population.

Our identification of differentially expressed genes included several genes that are subunits of protein phosphatase 1 (PP1), particularly *PPP1R11, PPP1R3B*, and *PPP1R18*. Previous studies have found that PP1 plays a role in dephosphorylating PP1 to reduce translation during some viral infections and recent studies have indicated a potential role in SARS-CoV-2 infections (He et al., 1997; Nekhai et al., 2007; Ilinykh et al., 2014; McDermott et al., 2016). Another gene found to be differentially expressed is *GSDMC*, whose protein product can be processed by caspase, which leads to pyroptosis (Liu et al., 2021). Pyroptosis has been observed in macrophages infected with SARS-CoV-2, which may be a factor that promotes the large inflammatory response associated with severe COVID-19 (Yap et al., 2020; Ferreira et al., 2021). Another differentially expressed gene is *DDX39B*, which is a mRNA splicing factor that plays a role in the nuclear export of mRNA (Fleckner et al., 1997; Zhang et al., 2021). It has also been found to play a role in inhibiting NF-kappaB (Szymura et al., 2020). Our study found that *DDX39B* was up-regulated in many of the sample conditions, which could be one of the methods the virus uses to avoid an inflammatory response after infection. Other studies have found that *DDX39B* can be targeted by the viral Nsp1 (Zhang et al., 2021) protein that serves to suppress the host immune response and decrease host gene expression so that cellular resources can be allocated to the translation of viral proteins (Yuan et al., 2021). A study of fatal COVID-19 patients found that *DDX39B* was significantly down regulated, which suggests this gene product may play a role in differentiating between mild and severe cases of COVID-19; particularly since the downregulation of *DDX39B* would be expected to trigger an inflammatory response that may get out of control in patients with severe COVID-19 (Wang et al., 2022).

We specifically chose our earliest time point to be 6hpi to give the virus sufficient time to affect the host cell. Although we were unable to directly quantify the presence of viral gene products in this study, is it known that viral proteins can affect the innate immune response (Shemesh et al., 2021; Vazquez et al., 2021; Cheemarla et al., 2021; Zhao et al., 2021) negatively impact the translation of host transcripts (Thoms et al., 2020; Simeoni et al., 2021). We assume the virus would produce relatively low levels of gene products prior to 6hpi, which would usually activate the interferon and other antiviral responses. However, the virus remains undetectable in alveolar macrophages (Dalskov et al., 2020), and may be only minimally detected in other cell types. This strategy enables the virus to produce its own components until the cell is overwhelmed and no longer able to contain the infection.

Our analysis of the GO enrichment results identified gene products associated with Zinc. Interestingly, recent work has shown that multiple host zinc metalloproteins interact with viral gene products including orf8 (Shemesh et al., 2021). In addition, Zinc has been shown to inhibit the viral protease so any decrease in host intracellular Zinc-related functions is likely biologically relevant (Vazquez et al., 2021; Cheemarla et al., 2021). Although the immune system component of infection has been well characterized (Thoms et al., 2020; Zhao et al., 2021), the modification of tight junctions and cell adhesion have also been associated with viral pathogenesis (Dalskov et al., 2020; Simeoni et al., 2021; Chasapis et al., 2021). In infections with the New York strain, we also see a negative regulation of terms that are involved in cholesterol biosynthesis. It has been shown that cholesterol plays an important role in many steps of the coronavirus life cycle and lower levels of cholesterol have been associated with increased COVID-19 severity and mortality (Panchariya et al., 2021; Grifagni et al., 2021; Ferrarini et al., 2021). We also see many GO terms that are related to the immune system and infection, which is to be expected. These include sets of genes that were observed to be affected in other experiments with SARS-CoV-2 in a few different cell types (Liang et al., 2020). Additional studies are needed to better understand the cellular and viral components that play a role in these functions.

The statistical signaling pathway analysis identified the plasminogen activating cascade as being inhibited at 6hpi and 12hpi. Although this pathway has been identified previously in the hamster model (Rauti et al., 2021), its directionality was not reported. The PDGFR pathway has been identified previously as decreasing SARS-CoV-2 replication (Cao et al., 2021). However, our NHBE cell model showed evidence that this pathway was positively affected 24hpi. We found it interesting that a small number of pathways could be modulated in opposite directions at the 12hpi and 24hpi time points for the NY strain. This may be a contributor to the increased pathogenesis associated with infections occurring in NY early in the pandemic. We were not surprised that infection with either virus isolate would affect 10 translation-related processes, since viruses take control of the host cellular machinery to generate genetic material, produce new viral proteins, and reduce host immune-related defenses (Raghavan et al., 2021; Zinellu et al., 2021; Masana et al., 2021; Dai et al., 2022).

Our validation of the previous finding that SARS-CoV-2 infection has a statistically significant effect on the actin cytoskeleton pathway was also of interest (Blanco-Melo et al., 2020; Suresh et al., 2021). At least two explanations for this observation are possible. The first could be that infected cells reorganize their cytoskeleton either in an attempt to prevent virus replication or as a mechanism for the virus to assemble virions more efficiently inside of the cell. The second possible explanation may only be relevant in the context of an infected human, where this pathway has been suggested to play a role in vascular structure in aging mice (Klann et al., 2020). It is possible that actin could at least partially explain some of the cardiovascular, neurological, and other signs and symptoms that are observed in a subset of infected patients (Belhadjer et al., 2020; Liu et al., 2020; Yuan et al., 2020; Alexander et al., 2021; Khatoon et al., 2021; Kim et al., 2021).

We found the observation that the SRP-dependent Cotranslational Protein Targeting to Membrane pathway was activated (NY strain) or inhibited (WA strain) to be of interest. This pathway has been reported previously in human samples, although the lineage(s) of the SARS-CoV-2 viruses were not consistently reported in these studies (Banerjee et al., 2020; Rivera et al., 2020; Daamen et al., 2021; Ibrahim and Ellakwa, 2021; Mohan and Wollert, 2021; Messina et al., 2021). A separate computational analysis used a machine learning approach to identify members of this same pathway to have high accuracy as markers of SARS-CoV-2 infection (Rabaglino et al., 2021).

It is important to recognize that the present study specifically focused on comparing the intracellular host transcriptional response to two viral isolates collected early in the pandemic. Additional experiments will be needed to better characterize how subsequent emergent SARS-CoV-2 variants may interact with, and affect, the host response and disease severity.

## Conclusion

This study provides additional insight into the intracellular host transcriptional response associated with either the New York or Washington SARS-CoV-2 isolates, which were collected in early 2020 when the pandemic was in its early stages. We identified genes, annotated functions, and signaling pathways that may be associated with the different pathogenicity profiles for these strains. We expect that these findings will be relevant to the development of improved prophylactics and/or therapeutics to minimize the underlying mechanistic effects of severe disease.

## Data availability statement

The datasets presented in this study can be found in online repositories. The names of the repository/repositories and accession number(s) can be found below: https://www.ncbi.nlm.nih.gov/geo/, GSE207923.

## Author contributions

TS contributed to data analysis, data interpretation, and manuscript preparation. AS-L and J.BL contributed laboratory experiments and manuscript review. RR and BB contributed experimental design, data interpretation and manuscript review. BP contributed inception, data analysis, data interpretation, code, and manuscript preparation. All authors contributed to the article and approved the submitted version.

## Acknowledgments

The following reagent was deposited by the Centers for Disease Control and Prevention and obtained through BEI Resources, NIAID, NIH: SARS-Related Coronavirus 2, Isolate USA-WA1/2020, NR-52281. The following reagent was obtained through BEI Resources, NIAID, NIH: SARS-Related Coronavirus 2, Isolate New York 1-PV02001/2020, NR-52368. We are grateful to the BYU Office of Research Computing for providing the computational resources needed to complete this study.

## Conflict of interest

The authors declare that the research was conducted in the absence of any commercial or financial relationships that could be construed as a potential conflict of interest.

## Publisher’s note

All claims expressed in this article are solely those of the authors and do not necessarily represent those of their affiliated organizations, or those of the publisher, the editors and the reviewers. Any product that may be evaluated in this article, or claim that may be made by its manufacturer, is not guaranteed or endorsed by the publisher.

## Supplementary material

Supplementary Figure 1 Pie charts of significant Gene Ontology functional terms across both strains at 6hpi and 12hpi.

Supplementary Table 1 Significant differentially expressed genes in the NY isolate (infected vs mock-infected) at 6hpi.

Supplementary Table 2 Significant differentially expressed genes in the NY isolate (infected vs mock-infected) at 12hpi.

Supplementary Table 3 Significant differentially expressed genes in the NY isolate (infected vs mock-infected) at 24hpi.

Supplementary Table 4 Significant differentially expressed genes in the WA isolate (infected vs mock-infected) at 6hpi.

Supplementary Table 5 Significant differentially expressed genes in the WA isolate (infected vs mock-infected) at 12hpi.

Supplementary Table 6 Significant differentially expressed genes in the WA isolate (infected vs mock-infected) at 24hpi.

Supplementary Table 7 Combined set of most significant differentially expressed genes across viral isolates and time points.

Supplementary Table 8 Significant affected functional terms in the NY isolate (infected vs mock-infected) at 6hpi.

Supplementary Table 9 Significant affected functional terms in the NY isolate (infected vs mock-infected) at 12hpi.

Supplementary Table 10 Significant affected functional terms in the NY isolate (infected vs mock-infected) at 24hpi.

Supplementary Table 11 Significant affected functional terms in the WA isolate (infected vs mock-infected) at 6hpi.

Supplementary Table 12 Significant affected functional terms in the WA isolate (infected vs mock-infected) at 12hpi.

Supplementary Table 13 Significant affected functional terms in the WA isolate (infected vs mock-infected) at 24hpi.

Supplementary Table 14 Significant affected signaling pathways in the NY isolate (infected vs mock-infected) at 6hpi.

Supplementary Table 15 Significant affected signaling pathways in the NY isolate (infected vs mock-infected) at 12hpi.

Supplementary Table 16 Significant affected signaling pathways in the NY isolate (infected vs mock-infected) at 24hpi.

Supplementary Table 17 Significant affected signaling pathways in the WA isolate (infected vs mock-infected) at 6hpi.

Supplementary Table 18 Significant affected signaling pathways in the WA isolate (infected vs mock-infected) at 12hpi.

Supplementary Table 19 Significant affected signaling pathways in the WA isolate (infected vs mock-infected) at 24hpi.
