# Supplementary material for: Comparison of Intracellular Transcriptional Response of NHBE Cells to Infection with SARS-CoV-2 Washington and New York Strains
Source: Front Cell Infect Microbiol. 2022 Sep 20;12:1009328. doi: 10.3389/fcimb.2022.1009328 (PMC9530606; doi:10.3389/fcimb.2022.1009328)
Supplement: Supplementary Figure 1 — Pie charts of significant Gene Ontology functional terms across both strains at 6hpi and 12hpi. [file Image_1.pdf]

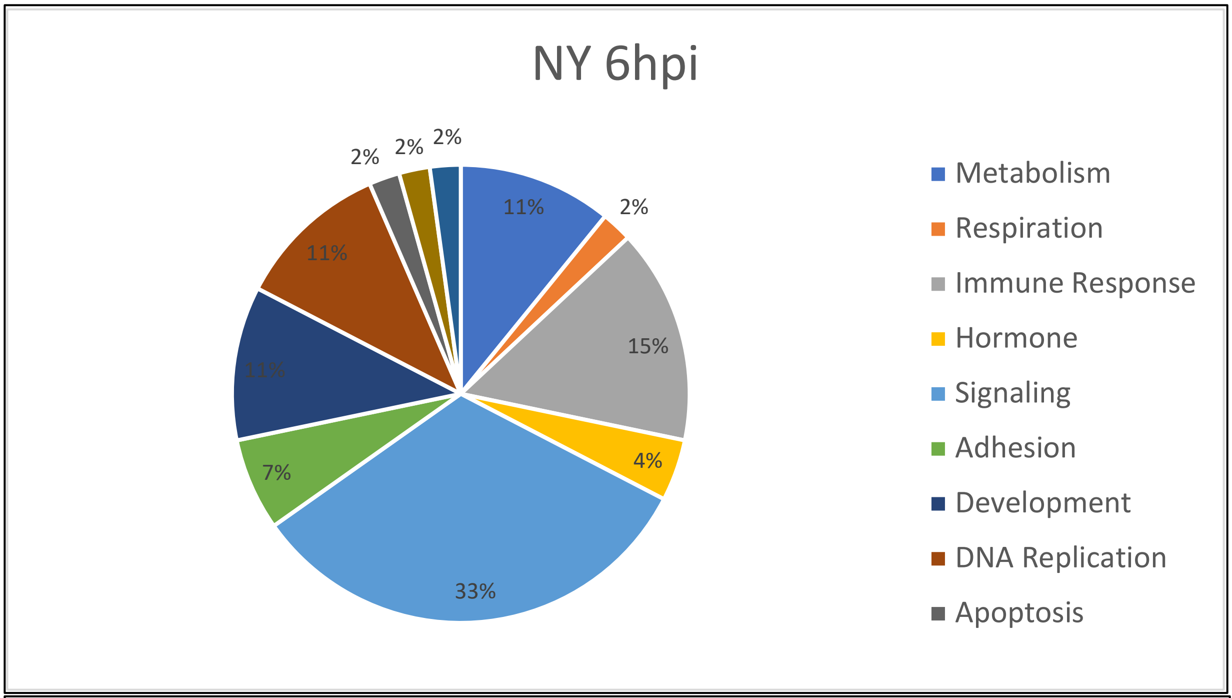
NY 6hpi
2%
2%
2%
11%
2%
11%
15%
11%
4%
7%
33%
Metabolism
Respiration
Immune Response
Hormone
Signaling
Adhesion
Development
DNA Replication
Apoptosis

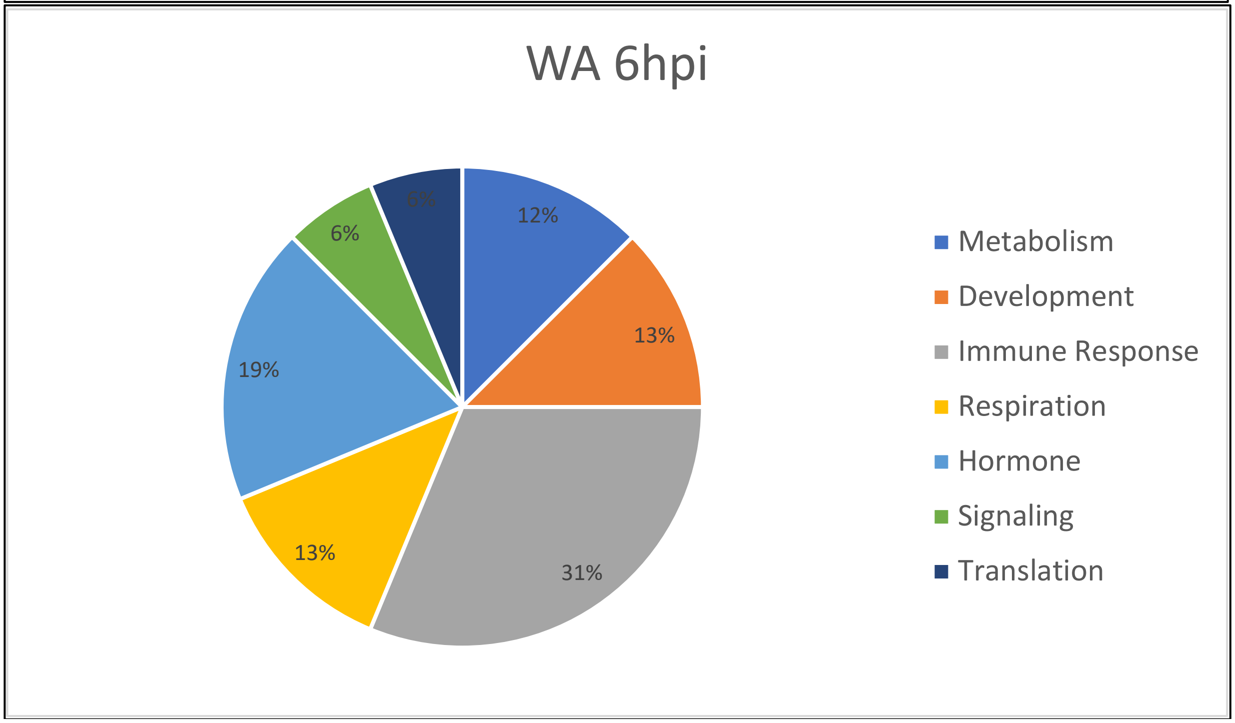
WA 6hpi
12%
6%
13%
19%
13%
31%
Metabolism
Development
Immune Response
Respiration
Hormone
Signaling
Translation

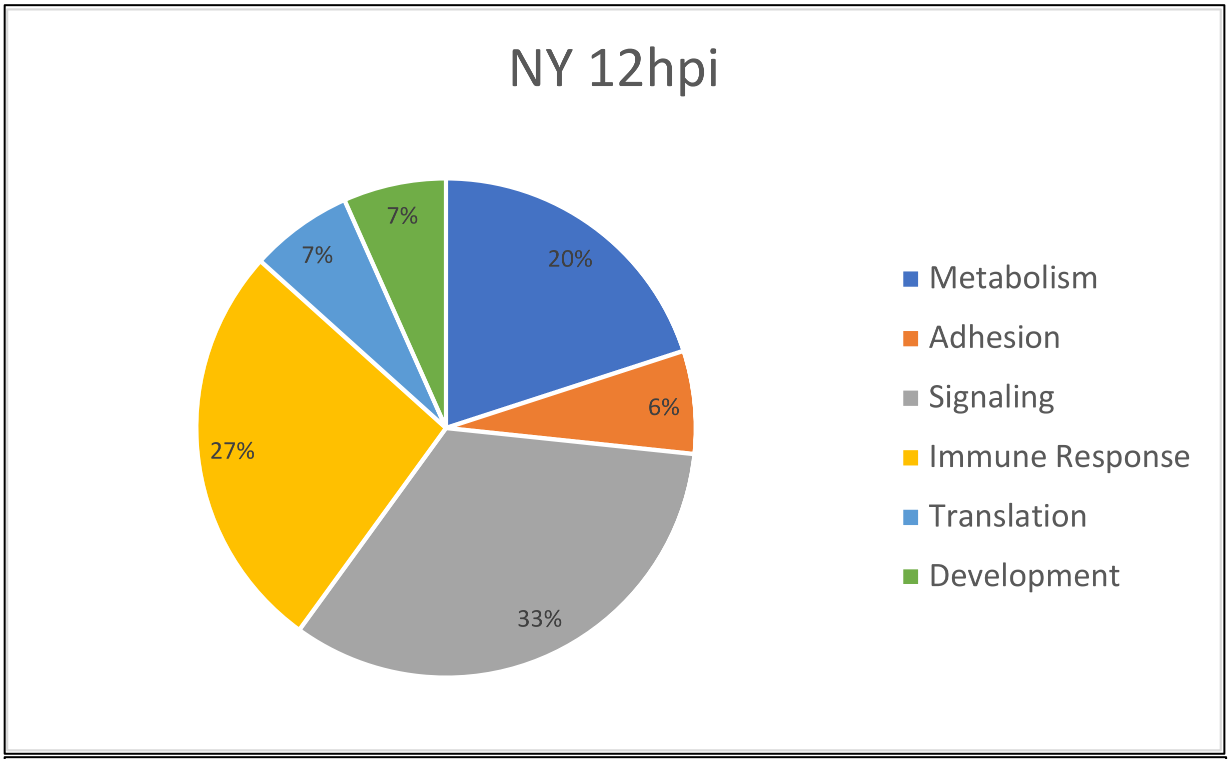
NY 12hpi
7%
7%
20%
6%
27%
33%
Metabolism
Adhesion
Signaling
Immune Response
Translation
Development

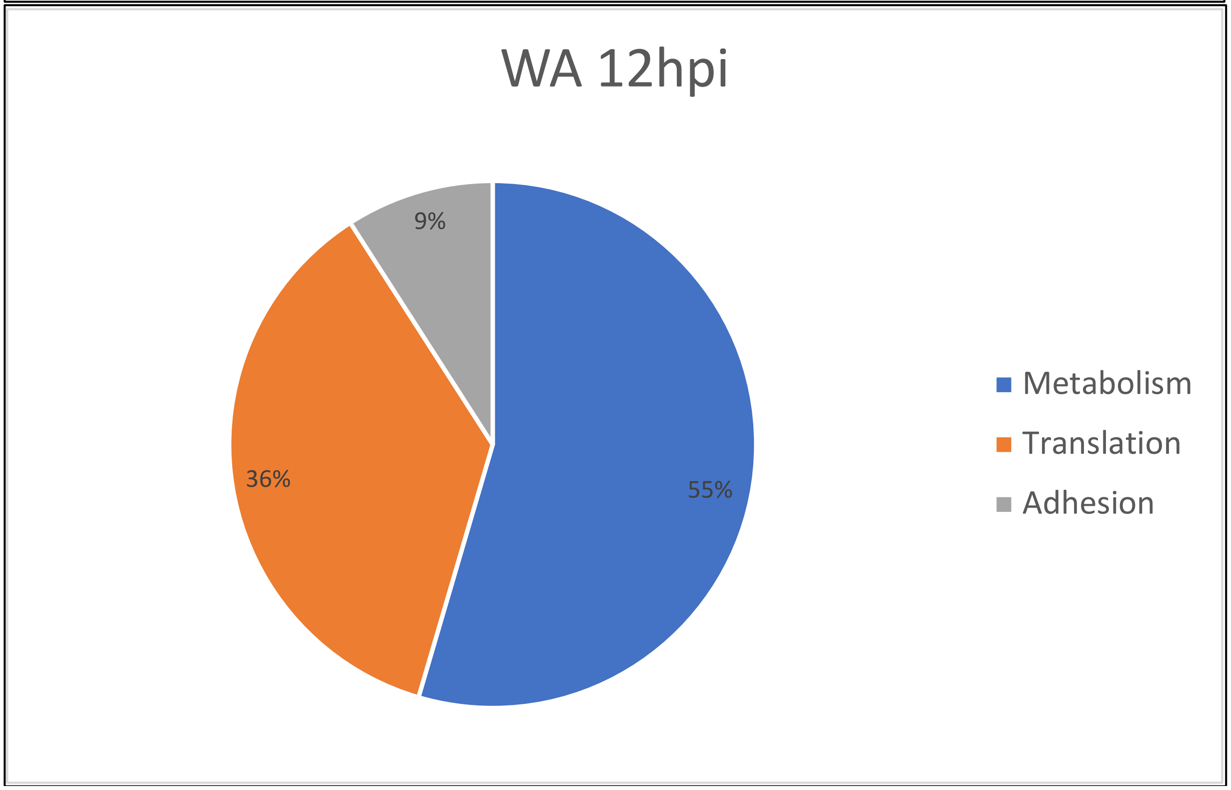
WA 12hpi
9%
36%
55%
Metabolism
Translation
Adhesion
